# IVIg and LPS Co-stimulation Induces IL-10 Production by Human Monocytes, Which Is Compromised by an FcγRIIA Disease-Associated Gene Variant

**DOI:** 10.3389/fimmu.2018.02676

**Published:** 2018-11-20

**Authors:** Lisa K. Kozicky, Susan C. Menzies, Zheng Yu Zhao, Tariq Vira, Kiera Harnden, Kwestan Safari, Kate L. Del Bel, Stuart E. Turvey, Laura M. Sly

**Affiliations:** ^1^Division of Gastroenterology, Department of Pediatrics, BC Children's Hospital and the University of British Columbia, Vancouver, BC, Canada; ^2^Division of Allergy and Immunology, Department of Pediatrics, BC Children's Hospital and the University of British Columbia, Vancouver, BC, Canada

**Keywords:** IVIg, IL-10, monocyte, MAPK, ERK, p38, FcγRIIA, rs1801274

## Abstract

Intravenous Immunoglobulin (IVIg) is used to treat autoimmune or inflammatory diseases, but its mechanism of action is not completely understood. We asked whether IVIg can induce interleukin-10 (IL-10) and reduce pro-inflammatory cytokine production in human monocytes, and whether this response is reduced in monocytes from people with an Fcγ receptor IIA (FcγRIIA) gene variant, which is associated with increased risk of inflammatory diseases and poor response to antibody-based biological therapy. IVIg increased IL-10 production and reduced pro-inflammatory cytokine production in response to bacterial lipopolysaccharide (LPS), which required FcγRI and FcγRIIB and activation of MAPKs, extracellular signal-regulated kinase 1/2 (ERK1/2), and p38. IL-10 production was lower and pro-inflammatory cytokine production was higher in monocytes from people with the FcγRIIA risk variant and the risk variant prevented IL-10 production in response to (IVIg+LPS). Finally, we show that IVIg did not induce MAPK activation in monocytes from people with the risk variant. Our results demonstrate that IVIg can skew human monocytes to an anti-inflammatory, IL-10-producing activation state, which is compromised in monocytes from people with the FcγRIIA risk variant. This research has profound implications for the use of IVIg because 25% of the population is homozygous for the FcγRIIA risk variant and its efficacy may be reduced in those individuals. In addition, this research may be useful to develop new therapeutic strategies to replace IVIg by cross-linking FcγRIs and FcγRIIBs to promote anti-inflammatory macrophage activation, independent of the FcγRIIA genotype.

## Introduction

Autoimmune and inflammatory diseases such as inflammatory bowel disease (IBD) and rheumatoid arthritis (RA) can result from imbalance between inflammation and its resolution. Defects in monocyte/macrophage function characterize many inflammatory diseases, which make them key therapeutic targets (1). Macrophages are phagocytic innate immune cells, well known for initiating inflammatory responses during infection or tissue injury, and directing the acquired immune response. They also play an important role in the resolution of inflammation actively suppressing the inflammatory response, by production of IL-10 (2).

IL-10 is a potent, non-redundant anti-inflammatory cytokine, which can limit inflammatory signaling from innate and adaptive immune cells (3). Importantly, dysregulation of IL-10 can result in the development of inflammatory diseases, such as IBD (4). Macrophages can adopt an anti-inflammatory activation state in which they secrete high amounts of IL-10 and dramatically lower amounts of pro-inflammatory cytokines in response to immune complexes (Ic) and stimuli that are normally pro-inflammatory (1).

Intravenous immunoglobulin (IVIg) is a drug made up of pooled polyclonal IgG antibodies, derived from the plasma of more than 1,000 blood donors. IVIg was originally used to supplement antibodies in people with antibody deficiencies, such as hypogammaglobinemia, or to supplement the immune system after bone marrow transplant (5). IVIg is also used to treat a wide variety of autoimmune and inflammatory diseases, such as idiopathic thrombocytopenic purpura (ITP), Kawasaki disease, chronic inflammatory demyelinating polyneuropathy (CIDP), and graft vs. host disease (5). Despite its broad use, the mechanism(s) of IVIg's anti-inflammatory activity is not completely understood (6). IVIg is given at very high doses (1–2 g/kg), and few proposed mechanisms explain this requirement (7). Although it has not been demonstrated in human studies, a leading theory is that a minor fraction of Fc portions of IgGs in IVIg are sialylated, which may be responsible for its efficacy, by up-regulating the inhibitory FcγRIIB (8–10).

The Fcγ receptor, FcγRIIA, has a relatively low affinity for IgG antibodies, and is found on the surface of myeloid cells and platelets (11). A gene variant for FcγRIIA (rs1801274) can change the receptor from a relatively low affinity to high affinity for binding IgG antibodies. The low affinity gene variant for FcγRIIA-R131, or CC genotype, has an arginine at amino acid 131 that confers a lower binding affinity for IgG1 and IgG3; whereas the FcγRIIA-H131, or TT genotype, has a histidine substituted at amino acid 131, which confers a higher binding affinity for IgG1 and IgG3 and confers binding affinity for IgG2 that is not present in the CC genotype (12–15). In a European population, the genotype frequencies are 28.3% CC, 45.1% CT, and 26.6% TT (dbSNP). The disease-associated gene variant (TT) has been associated with an increased risk of inflammatory diseases in people, including ulcerative colitis and Kawasaki disease (16–18). It is also associated with an increased risk of failure to respond to therapy with the anti-TNFα antibody, infliximab, in people with RA (12).

Our laboratory has found that mouse bone marrow-derived and peritoneal macrophages can be activated to produce high levels of IL-10 and low levels of IL-12/23p40, similar to macrophages stimulated with Ic and bacterial lipopolysaccharide (LPS), M(Ic + LPS). To investigate this in human cells, we asked whether human monocytes have increased IL-10 and decreased pro-inflammatory cytokine production when stimulated with (IVIg + LPS), similar to mouse macrophages. We also asked whether monocytes from people with the FcγRIIA risk variant have compromised ability to induce anti-inflammatory IL-10-producing monocytes in response to IVIg, which could explain the increased risk of inflammatory diseases and failure to respond to infliximab that are associated with the risk variant.

Herein, we report a novel mechanism by which IVIg reduces inflammatory responses in human monocytes. Moreover, we report that monocytes from people with the FcγRIIA disease-associated gene variant have compromised ability to reduce inflammatory cytokine production in response to IVIg. (IVIg + LPS) activated monocytes produce higher levels of anti-inflammatory IL-10 and lower levels of pro-inflammatory cytokines IL-12/23p40, IL-6, and TNF. FcγRI and FcγRIIB, but not FcγRIIA or FcγRIII, are required for IVIg-induced IL-10 production in human monocytes and activation of the MAPKs, p38 and ERK1/2, is required for IVIg-induction of IL-10. IL-10 signaling contributes to reduced LPS-induced pro-inflammatory cytokine production by (IVIg + LPS)-treated monocytes. Moreover, we show that monocytes from people with the FcγRIIA risk variant have lower IVIg-mediated anti-inflammatory responses, including lower IL-10 production and higher pro-inflammatory cytokine production compared to monocytes from people with the non-risk variant. Importantly, we confirm specifically that the FcγRIIA risk variant is responsible for preventing IVIg-induced IL-10 production. Finally, we show that IVIg co-stimulation does not activate MAPKs in monocytes from people with the risk variant. Taken together, these results demonstrate that IVIg can skew human monocytes to an anti-inflammatory IL-10-producing activation state, and anti-inflammatory activation is compromised in monocytes from people with the high affinity, disease-associated FcγRIIA gene variant. These new data may be useful in guiding the selection of patient populations most likely to respond to IVIg and may inform the development of novel anti-inflammatory therapies.

## Materials and methods

### Monocyte isolation and culture

PBMCs were isolated from healthy control blood by density gradient centrifugation, using Lymphoprep (STEM CELL Technologies, Vancouver, BC, CA). Cells were washed and suspended in Iscove's Modified Dulbecco's Medium (IMDM) supplemented with 10% autologous serum and penicillin/streptomycin at a density of 2.0 × 10^6^/ml for 1.5 h. Non-adherent cells were washed away and adherent monocytes were re-plated at a density of 2.5 × 10^5^ cells/ml for 24 h before use in assays.

### Cell stimulations

Cells were plated at a density of 2.5 × 10^5^ cells/ml (100 μl/well in a 96-well plates) were left unstimulated or stimulated with 100 ng/ml LPS (*Escherichia coli* serotype 127:B8; Sigma-Aldrich, St. Louis, MO, USA), 5 mg/ml IVIg (Gamunex Immune Globulin Intravenous 10% solution for infusion; Transfusion Medicine, BC Children's Hospital, Vancouver, BC, CA), or both IVIg + LPS. After incubation, cell supernatants were harvested and clarified by centrifugation for analyses. For Fcγ receptor blocking experiments, antibodies were added 1 h prior to stimulations, at final concentrations of: IgG isotype control antibody (50 or 100 μg/ml ; AB-108-C, R & D Biosystems, Minneapolis, MN, USA), FcγRI blocking antibody (100 μg/ml, AF 1257, R & D Biosystems), FcγRIIA blocking antibody (50 μg/ml, AF 1875, R & D Biosystems), FcγRIIB/C blocking antibody (100 μg/ml, AF 1330, R & D Biosystems), and FcγRIII blocking antibody (50 μg/ml, AF 1597, R & D Biosystems). For IL-10 experiments, recombinant human IL-10 (rhIL-10; STEM CELL Technologies) was added at a final concentration of 400 pg/ml. For IL-10 receptor blocking experiments, antibodies were added 1 h prior to stimulations, at final concentrations of 5 μg/ml for both the IgG isotype control antibody (clone RTK2758 BioLegend, San Diego, CA, USA) or IL-10 receptor blocking antibody (clone 3F9 BioLegend). For inhibitor studies, inhibitors were added 1 h prior to stimulations, at final concentrations of: DMSO (vehicle control; 0.1%), PD98059 (50 μm, Cell Signaling Technology), SCH772984 (1 μm, MedChem Express, Princeton, NJ, USA), SB203580 (10 μm, Cell Signaling Technology, Danvers, MA, USA), or BIRB-796 (180 nm, Cayman Chemical, Ann Arbor, MI, USA),

### Cytokine measurements

Cytokines were assayed by ELISA, according to the manufacturer's instructions. ELISA kits for human IL-10, IL-12/23p40, IL-6, and TNF were from BD Biosciences (Mississauga, ON, Canada).

### SDS-PAGE and western blotting

Monocytes were stimulated for 0, 10, 40, or 120 min, as indicated. After stimulation, monocytes were placed on ice and rinsed twice with cold PBS. Whole cell lysates were prepared for SDS-PAGE by lysing in 1 × Laemmli's digestion mix, DNA was sheered using a 26-guage needle, and samples were boiled for 1 min. Cell lysates were separated on a 10% polyacrylamide gel and western blotting was carried out, as described previously (19). Antibodies used for western blot analyses for MAPK activation experiments were anti-pERK1/2 (Cell Signaling Technology, 9,106), anti-pp38 (Cell Signaling Technology, 4,631), and anti-GAPDH (Fitzgerald Industries International, 10R-G109a, Acton, MA, USA). Antibodies used for western blot analyses for siRNA experiments were anti-FcγRI (AbCam, ab119843, Cambridge, UK), anti-FcγRIIB (AbCam, ab151497), anti-FcγRIII (AbCam, ab94773), anti-FcγRIIA (AbCam, ab167381), anti-β-actin (Cell Signaling Technology, 4,970), anti-ERK1/2 (Cell Signaling Technology, 9,102), anti-p38 (Cell Signaling Technology, 9,212), and anti-GAPDH (Fitzgerald Industries International, 10R-G109a, Acton, MA, USA).

### Fcγ receptor and MAPK siRNA

Monocytes were untreated (UnRx) for 48 h or pre-treated for 48 h with siRNAs using Lipofectamine RNAiMAX reagent (Thermo Fischer, MA, USA) with 10 nm of a non-silencing siRNA (ns; silencer select negative control siRNA #1,Thermo Fischer) or 2 different silencer select siRNAs (si1 or si2) to the FcγRI (s5069 and s5070, Thermo Fischer), FcγRIIA (s194408 and s223525, Thermo Fischer), FcγRIIB (s5073 and s5075, Thermo Fischer), or FcγRIIIA (s57398 and s223526, Thermo Fischer). For MAPK siRNA experiments, 30 nm of a non-silencing siRNA (ns; silencer select negative control siRNA #1,Thermo Fischer) or 30 nm combined of different silencer select siRNAs (si1 or si2) to ERK1 (s11141 and s11142, Thermo Fischer) and ERK2 (s11137 and s11138, Thermo Fischer) or to p38α (s3585 and s3586, Thermo Fischer), p38γ (s12467 and s12468, Thermo Fischer), and p38δ (s11165 and s11166, Thermo Fischer). Cells were harvested for western blot analyses or stimulated and cell free supernatants were assayed for cytokines, as described above.

### Genotyping the FCGR2A inflammatory disease susceptibility SNP, rs1801274

Blood samples were frozen at −20°C, and DNA was extracted using a commercially available kit, according to manufacturer's instructions (QIAGEN, Hilden, Germany). DNA was used to genotype the FCGR2A SNP, rs1801274, using a commercially available Taqman assay C_9077561_20; Thermo Fischer Scientific, Waltham, MA, USA). SNPs were considered acceptable for analysis if they had call rates > 95% and frequencies did not deviate from Hardy-Weinberg equilibrium (*p* < 0.05). Analyses were performed on participants before stratification by genotype.

### Statistical analyses

Parametric or non-parametric unpaired 2-tailed *t*-tests or repeated measures one way ANOVAs with Dunn's multiple comparisons correction were used, where indicated. Graphpad prism software version 6.03 was used for analyses. Differences were considered significant at *p* < 0.05.

### Human ethics approval

All experimental procedures were performed in accordance with ethical guidelines and approved by the University of British Columbia research ethics board (H13-03524 and H14-00622). All subjects provided written consent for blood collection for immune cell isolation and functional assays, DNA isolation, and genotyping.

## Results

### IVIg increases IL-10 production and reduces pro-inflammatory cytokine production by human monocytes stimulated with LPS

**Figure 1:**
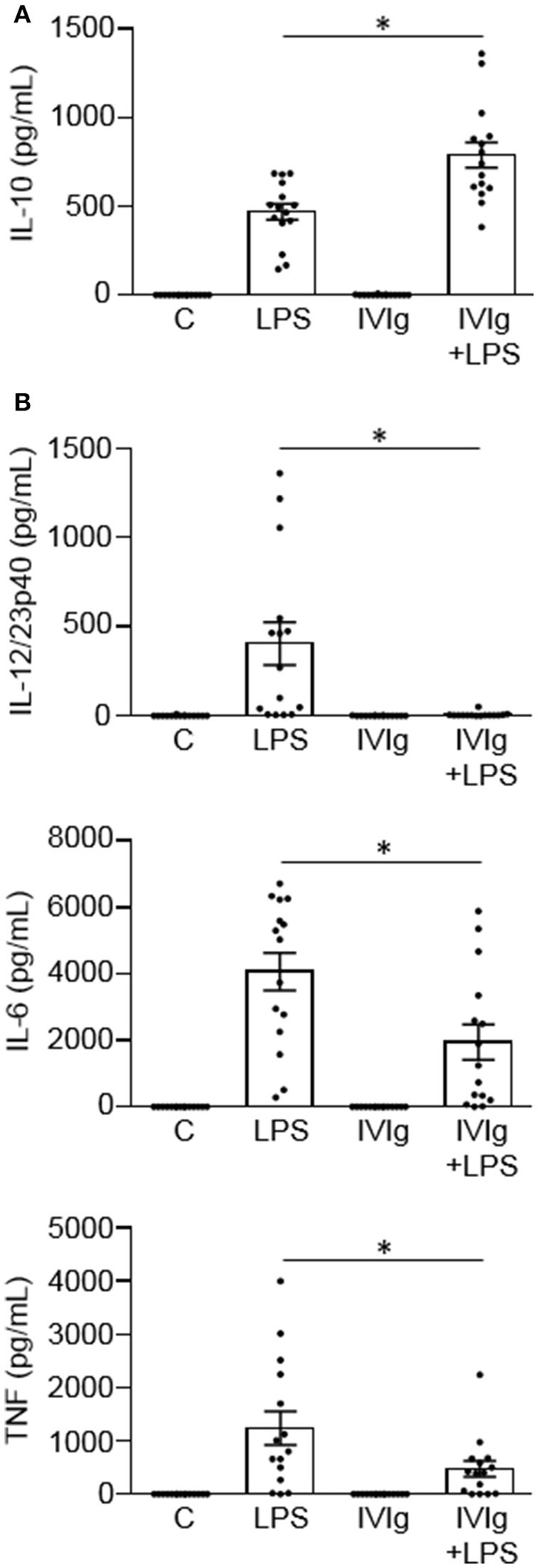
IVIg increases IL-10 production and reduces pro-inflammatory cytokine production in LPS-stimulated human monocytes. Monocytes from healthy control participants were unstimulated [Control (C)] or stimulated with LPS (100 ng/ml), IVIg (5 mg/ml), or both, for 24 h. Clarified cell supernatants were assayed for **(A)** IL-10, **(B)** IL-12/23p40, IL-6, or TNF by ELISA. Data are mean ± SEM with *n* = 16 participants performed as independent experiments, and assayed in duplicate. ^*^p < 0.001 for cells treated with LPS compared to cells treated with (IVIg + LPS). Statistical analyses were performed using a non-parametric paired *t*-test.

We have previously reported that IVIg-activated mouse bone marrow derived macrophages produce high levels of the anti-inflammatory cytokine IL-10 and low levels of pro-inflammatory cytokines, in response to the inflammatory stimulus, LPS (20). Herein, we asked whether IVIg-activated human monocytes [henceforth referred to as M(IVIg)] also produce more IL-10 and less pro-inflammatory cytokines in response to LPS. Peripheral blood monocytes from healthy control participants were unstimulated [Control (C)] or stimulated with LPS, IVIg, or (IVIg + LPS). Unstimulated or IVIg alone stimulated monocytes did not produce IL-10 (Figure 1A). LPS treatment induced modest amounts of IL-10, whereas (IVIg + LPS) treatment caused a 69% increase in IL-10 production (Figure 1A). Unstimulated or IVIg stimulated monocytes did not produce IL-12/23p40, IL-6, or TNF (Figure 1B). LPS induced high levels of IL-12/23p40 production, which was reduced by 98% when monocytes were co-stimulated with IVIg (Figure 1B). Similarly, IL-6 and TNF were produced by LPS treated monocytes and their production was significantly reduced, 52 and 62%, respectively, by concomitant treatment with IVIg (Figure 1B). These results show that co-stimulation with IVIg and LPS activates human monocytes to produce anti-inflammatory IL-10 and reduces pro-inflammatory cytokine production relative to LPS stimulation alone.

### FcγRI and FcγRIIB are required for IVIg-induced IL-10 production in response to LPS

**Figure 2:**
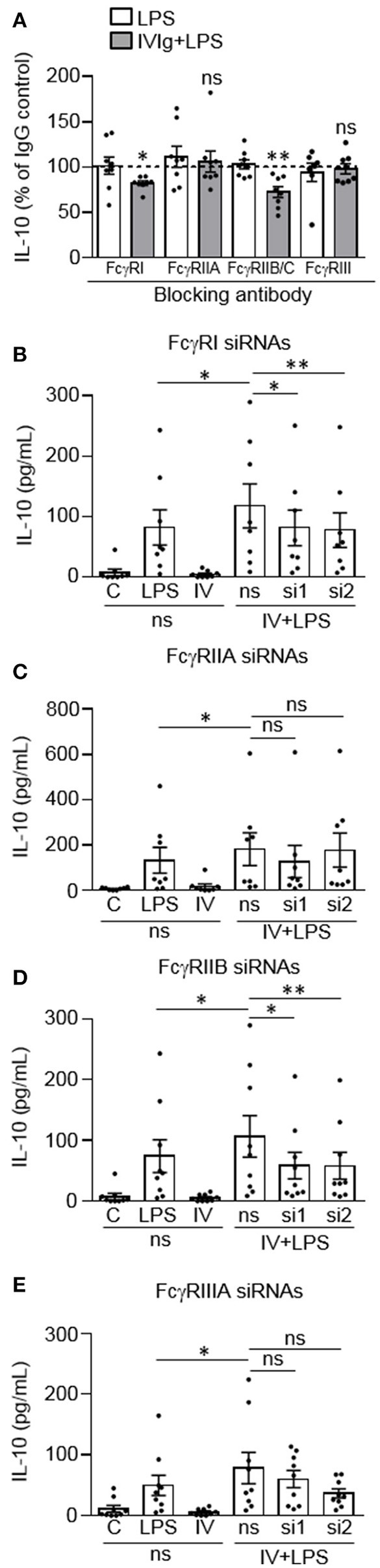
FcγRI and FcγRIIB are required for IVIg-induced IL-10 production in response to LPS. **(A)** Monocytes were untreated or pre-treated for 1 h with an IgG isotype control (50 or 100 μg/ml) or a blocking antibody against FcγRI (100 μg/ml), FcγRIIA (50 μg/ml), FcγRIIB/C (100 μg/ml), or FcγRIII (50 μg/ml). Cells were stimulated with LPS (100 ng/ml) or [IVIg (5 mg/ml) + LPS (100 ng/ml)] for 24 h. Clarified cell supernatants were assayed for IL-10. Statistical comparisons are for the IgG control to specific FcγR blocking antibody (raw data). **(B–E)** Monocytes were untreated or pre-treated for 48 h with a non-silencing siRNA (ns) or 2 different siRNAs (si1 or si2) to FcγRI **(B)**, FcγRIIA **(C)**, FcγRIIB **(D)**, or FcγRIIIA **(E)**. Monocytes pre-treated with the ns siRNA control were unstimulated [control (C)] or stimulated with LPS (100 ng/ml), IVIg (5 mg/ml), or both, for 24 h. Monocytes pre-treated with si1 or si2 were stimulated with IVIg (5 mg/ml) + LPS (100 ng/ml). Clarified cell supernatants were assayed for IL-10. Data are mean ± SEM. Results are representative of *n* = 8 experiments in **(A)** and *n* = 8 or 9 experiments in **(B–E)**; Monocytes were derived from 1 participant for each of 8 or 9 independent experiments, and were assayed in duplicate. ^*^p < 0.05, ^**^p < 0.01 and ns = not statistically different. Statistical analyses were performed using a repeated measures one-way ANOVA with Dunn's multiple comparisons correction.

We next wanted to determine which Fcγ receptor(s) are required for IL-10 production by (IVIg + LPS)-activated monocytes. Monocytes were left untreated or pre-treated blocking antibody to FcγRI, FcγRIIA, FcγRIIB/C, or FcγRIII, or an isotype control antibody (Figure 2A). Monocytes were then stimulated with LPS or (IVIg + LPS). LPS-induced IL-10 production was not affected when either FcγRI, FcγRIIA, FcγRIIB/C, or FcγRIII were blocked (white bars; Figure 2A). However, when stimulated with (IVIg + LPS), blocking FcγRI or FcγRIIB/C significantly decreased IL-10 production relative to the IgG control (82% of IgG control for FcγRI, 73% of IgG control for FcγRIIB/C; Figure 2A. There was no change in (IVIg + LPS)-induced IL-10 production when FcγRIIA or FcγRIII was blocked (Figure 2A). Raw data are provided in Supplemental Figure 1.

To confirm these findings, we used siRNAs specific to FcγRI, FcγRIIA, FcγRIIB, or FcγRIIIA, to determine the effect of receptor knockdown on (IVIg + LPS)-induced IL-10 production. Monocytes were untreated or pre-treated with a non-silencing siRNA control (ns) or two siRNAs (si1 and si2) to FcγRI (Figure 2B), FcγRIIA (Figure 2C), FcγRIIB (Figure 2D), or FcγRIIIA (Figure 2E). Cell lysates were prepared, separated by SDS-PAGE and analyzed by western blotting using antibodies for the FcγRI, FcγRIIA, FcγRIIB, or FcγRIIIA as well as β-actin, as a loading control. Representative western blots and quantification of knockdown are shown in Supplemental Figure 2. Monocytes were left unstimulated or stimulated with LPS, IVIg, or (IVIg + LPS), and IL-10 production was measured by ELISA. The untreated (UnRx) and ns siRNA had similar levels of FcγRI. The siRNA 1 and 2 each knocked down the FcγRI by 40% compared to the ns siRNA control (Supplemental Figure 2A). (IVIg + LPS)-induced IL-10 production was reduced significantly by siRNA1 and by siRNA2, compared to the ns siRNA control (31% siRNA1, 34% siRNA2; Figure 2B). For FcγRIIA, ns siRNA did not affect receptor expression. Compared to the ns siRNA control, the siRNA 1, and 2 reduced the FcγRIIA receptor expression by 56 and 46%, respectively (Supplemental Figure 2B). (IVIg + LPS)-induced IL-10 production was not reduced significantly during FcγRIIA knockdown with either siRNA1 or siRNA2 (*p* = 0.99 and *p* = 0.09; Figure 2C). Untreated and ns siRNA had similar levels of FcγRIIB. The siRNA 1 and 2 knocked down the FcγRIIB receptor by 58 and 60%, respectively, compared to the ns siRNA control (Supplemental Figure 2C). Compared to the ns siRNA control, (IVIg + LPS)-induced IL-10 production was reduced significantly by siRNA1 and by siRNA2 to FcγRIIB (45% siRNA1 and siRNA2; Figure 2D). For FcγRIIIA, ns siRNA did not affect receptor expression. Compared to the ns siRNA control, the siRNA 1 and 2 reduced the FcγRIIIA receptor expression by 44 and 51%, respectively (Supplemental Figure 2D). (IVIg + LPS)-induced IL-10 production was not reduced significantly during FcγRIIIA knockdown with either siRNA1 or siRNA2 (*p* = 0.96 and *p* = 0.07; Figure 2E). The reduction of LPS-induced pro-inflammatory cytokines IL-12/23p40, IL-6, and TNF by IVIg was not affected by siRNA knockdown of FcγRI, FcγRIIA, FcγRIIB, or FcγRIIIA (Supplemental Figure 3). Taken together, these results suggest that FcγRI and FcγRIIB are important for the induction of IL-10 in response to IVIg.

### MAPK signaling is required for IVIg-induced IL-10 production in response to LPS in human monocytes

**Figure 3:**
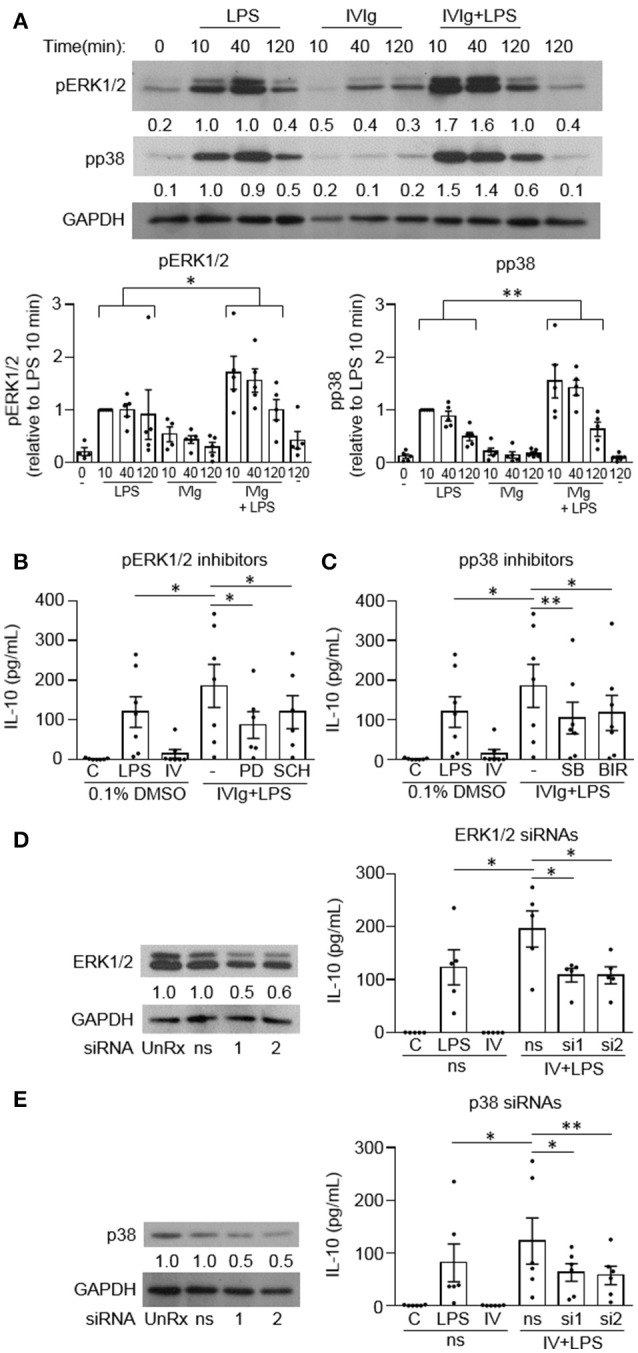
MAPKs are required for IVIg-induced IL-10 production in response to LPS. **(A)** Monocytes from healthy control participants were unstimulated or stimulated with LPS (100 ng/ml), IVIg (5 mg/ml), or both, for 0, 10, 40, or 120 min. Cell lysates (2.5 × 10^5^ cells / time point) were prepared at the indicated times. Lysates were separated by SDS-PAGE and analyzed by western blotting using phosphospecific antibodies for ERK1/2 and p38, and GAPDH, as a loading control. Results are representative of *n* = 5 experiments; monocytes were derived from 1 participant for each of 5 independent experiments. Densitometry for pERK1/2 and pp38; normalized to GAPDH and relative to LPS 10 min; are averaged and shown below each band and values are graphed and reported as mean ± SEM. In **(B)** and **(C)**, monocytes were pre-treated for 1 h with an appropriate volume of DMSO, as a vehicle control, or **(B)** the ERK1/2 inhibitors, PD98059 (PD) and SCH772984 (SCH), or **(C)** the p38 inhibitors, SB203580 (SB), and BIRB796 (BIR). In **(D)** and **(E)** monocytes were untreated (UnRx) or pre-treated for 48 h with a non-silencing siRNA (ns) or 2 different sets of siRNAs (si1 or si2) to ERK1 and 2 **(D)** or p38α, p38γ, and p38δ **(E)**. Samples in **(D)** and **(E)** were prepared, as above, and analyzed by western blotting using antibodies for ERK1/2 **(D)** or p38 **(E)** and GAPDH, as a loading control. Densitometry for ERK1/2 **(D)** or p38 **(E)** normalized to GAPDH and relative to untreated control (UnRx) are shown below each band. Monocytes in **(B–E)** were unstimulated **(C)** or stimulated with LPS (100 ng/ml), IVIg (5 mg/ml), or (IVIg + LPS) for 24 h. Clarified cell supernatants were assayed for IL-10 by ELISA. Values are reported as mean ± SEM for *n* = 7 **(B,C)** or *n* = 5 or 6 **(D,E)** participants performed as independent experiments, assayed in duplicate. ^*^p < 0.05, ^**^p < 0.01, and ns = not statistically different for the comparisons indicated. Statistical analyses were performed using a two-way ANOVA in **(A)** and repeated measures one-way ANOVA in **(B–E)** with Dunn's multiple comparisons correction.

Since mouse M(IVIg + LPS) and M(Ic + LPS) require MAPK signaling for IVIg- or Ic-induced IL-10 production in response to LPS, we next asked whether human M(IVIg) required MAPKs for IL-10 production in response to LPS (20, 21). Monocytes were unstimulated or stimulated with LPS (100 ng/ml), IVIg (5 mg/ml), or both for 0, 10, 40, or 120 min. Cell lysates were prepared at the indicated times, separated by SDS-PAGE, and analyzed by western blotting using phosphospecific antibodies for ERK1/2 and p38, and GAPDH, as a loading control (Figure 3A). LPS stimulation increased ERK1/2 activation compared to unstimulated cells. pERK1/2 was increased modestly with IVIg alone compared to unstimulated cells. (IVIg + LPS)-activated monocytes had significantly higher and prolonged ERK1/2 activation compared to LPS, with 1.7, 1.6, and 1.8-fold increases at 10, 40, and 120 min, respectively. LPS stimulation increased p38 activation compared to unstimulated cells. IVIg alone had little impact on pp38 levels compared to 0 and 120 min unstimulated controls. (IVIg + LPS) induced significantly higher and prolonged p38 activation compared to LPS. (IVIg + LPS)-induced pp38 was increased 1.5, 1.7 and 1.8-fold at 10, 40, and 120 min, respectively, compared to LPS-induced pp38 at each time point (Figure 3A).

To investigate the requirement for MAPK signaling on (IVIg + LPS)-induced IL-10 production, we used two independent pharmacological inhibitors to each MAPKs, ERK1/2 and p38. PD98059 (PD) inhibits the activation of MEK1, the ERK1/2 kinase and SCH772984 (SCH) is a novel ERK1/2 inhibitor. PD98059 significantly reduced IL-10 production in response to (IVIg + LPS) compared to the DMSO (vehicle) control (53%; Figure 3B). SCH772984 also reduced IL-10 significantly (35%; Figure 3B). SB203580 (SB) and BIRB796 (BIR) are potent and selective p38 inhibitors; SB203580 inhibits p38α and p38β, while BIRB796 inhibits p38α. Compared to the solvent control (DMSO), both SB203580 and BIRB796 significantly reduced (IVIg + LPS)-induced IL-10 production (43% SB, 36% BIR; Figure 3C).

To complement our inhibitor experiments, we also performed siRNA knockdown of ERK1/2 and p38. Monocytes were untreated (UnRx) or pre-treated with a non-silencing siRNA control (ns) or two sets of siRNAs to ERK1 and 2 (si1 and si2; Figure 3D) or to p38α, p38γ, and p38δ, which are the p38 isotypes expressed in human monocytes (Figure 3E) (22). Cell lysates were prepared, separated by SDS-PAGE, and analyzed by western blotting by probing with antibodies for ERK1/2 or p38 and GAPDH, as a loading control. Representative western blots and quantifications of protein expression are shown (Figures 3D,E, left). Monocytes were left unstimulated or stimulated with LPS, IVIg, or (IVIg + LPS), and IL-10 production was measured by ELISA. The untreated (UnRx) and ns siRNA had similar levels of ERK1/2. The siRNA 1 and 2 each knocked down ERK1/2 by 56 and 59% compared to the ns siRNA control (Figure 3D). (IVIg + LPS)-induced IL-10 production was reduced significantly by siRNA1 and by siRNA2, compared to the ns siRNA control (44% siRNA1, 45% siRNA2; Figure 3D). The untreated (UnRx) and ns siRNA had similar levels of p38. The siRNA 1 and 2 each knocked down p38 by 52 and 53% compared to the ns siRNA control (Figure 3E). (IVIg + LPS)-induced IL-10 production was reduced significantly by siRNA1 and by siRNA2, compared to the ns siRNA control (48% siRNA1, 53% siRNA2; Figure 3E). These results demonstrate that the MAPKs p38 and ERK1/2 are required for (IVIg + LPS)-induced IL-10 production.

### IL-10 signaling reduces pro-inflammatory cytokine production by (IVIg+LPS)-activated monocytes

**Figure 4:**
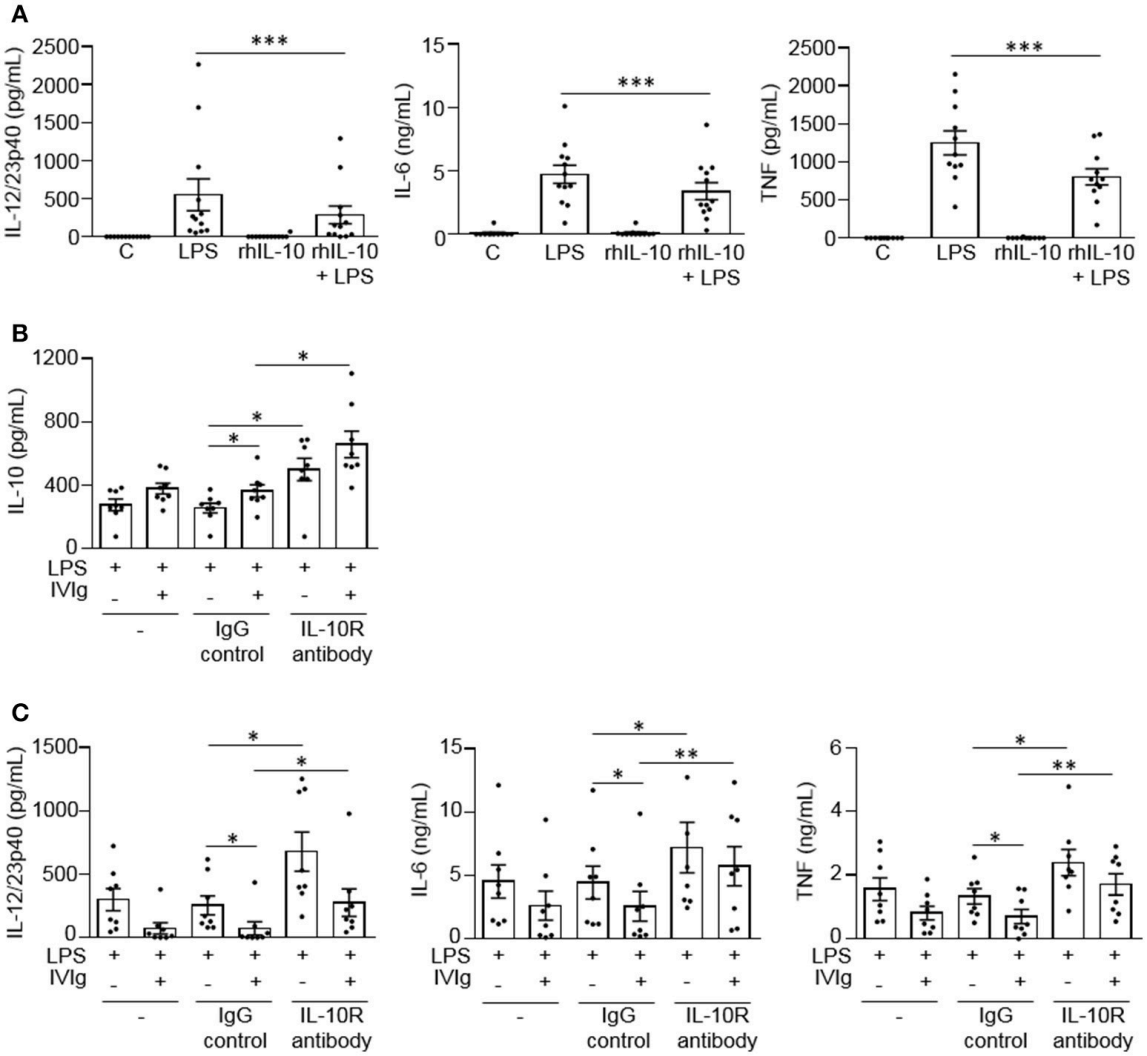
IL-10 signaling contributes to reduced LPS-induced pro-inflammatory cytokine production by IVIg-activated monocytes. In **(A)** monocytes from healthy control participants were left untreated **(C)** or stimulated with LPS (100 ng/ml), recombinant human IL-10 (rhIL-10; 400 pg/ml) or [rhIL-10 (400 pg/ml) + LPS (100 ng/ml)] for 24 h. Clarified cell supernatants were assayed for **(A)** IL-12/23p40, IL-6, and TNF. In **(B,C)** monocytes from healthy control participants were untreated (–) or pre-treated for 1 h with an IgG isotype control (IgG; 5 μg/ml) or an IL-10 receptor (IL-10R) blocking antibody (5 μg/ml). Cells were then stimulated with LPS (100 ng/ml) or [IVIg (5 mg/ml) + LPS (100 ng/ml)] for 24 h. Clarified cell supernatants were assayed for **(B)** IL-10, **(C)** IL-12/23p40, IL-6, and TNF. Data are mean ± SEM from *n* = 12 **(A)** or 8 **(B,C)** participants performed as independent experiments, assayed in duplicate. ^*^p < 0.05 and ^**^p < 0.01, and ^***^p < 0.001 for the comparisons indicated. Statistical analyses were performed using a non-parametric paired *t*-test in **(A)** and a repeated measures one-way ANOVA with Dunn's multiple comparisons correction in **(B,C)**.

To determine whether IL-10 can contribute to the reduction of LPS-induced pro-inflammatory cytokine production, we co-stimulated monocytes with recombinant human IL-10 (rhIL-10) and LPS. Cells were either unstimulated [control (C)] or stimulated with LPS, rhIL-10, or (rhIL-10 + LPS). Pro-inflammatory cytokines produced were measured in cell supernatants by ELISA. The unstimulated control or rhIL-10 alone did not cause cytokine production. rhIL-10 significantly decreased production of LPS-induced IL-12/23p40, IL-6, and TNF by monocytes (48, 28, and 36%, respectively; Figure 4A).

To determine whether IL-10 signaling contributes to lower pro-inflammatory cytokine production by (IVIg + LPS)-activated monocytes, we blocked IL-10 signaling with a blocking antibody against the IL-10 receptor during stimulation. Cells were either untreated or pre-treated with an IgG isotype control antibody (IgG) or an anti-IL-10 receptor blocking antibody (IL-10R antibody), and stimulated with LPS or (IVIg + LPS). IL-10 and pro-inflammatory cytokines production were measured in cell supernatants by ELISA. For all treatments, the unstimulated control or IVIg alone did not cause cytokine production (data not shown). Monocytes pre-treated with the IL-10 receptor blocking antibody produced significantly more IL-10 when stimulated with LPS or (IVIg + LPS) compared to antibody control-treated monocytes (94 and 81%, respectively; Figure 4B). IL-10 receptor blockade increased pro-inflammatory cytokine production in LPS and (IVIg + LPS)-stimulated monocytes compared to the IgG control. In response to LPS or (IVIg + LPS) stimulation, respectively, IL-12/23p40 production increased 165 and 278% (Figure 4C), IL-6 production increased 62 and 123% (Figure 4C), and TNF production increased 80 and 144% (Figure 4C). These results suggest that IL-10 production contributes to the reduction of pro-inflammatory cytokines produced in response to (IVIg + LPS).

### IVIg-induced anti-inflammatory macrophage activation is lower in monocytes from people with the high affinity FcγRIIA risk variant

**Figure 5:**
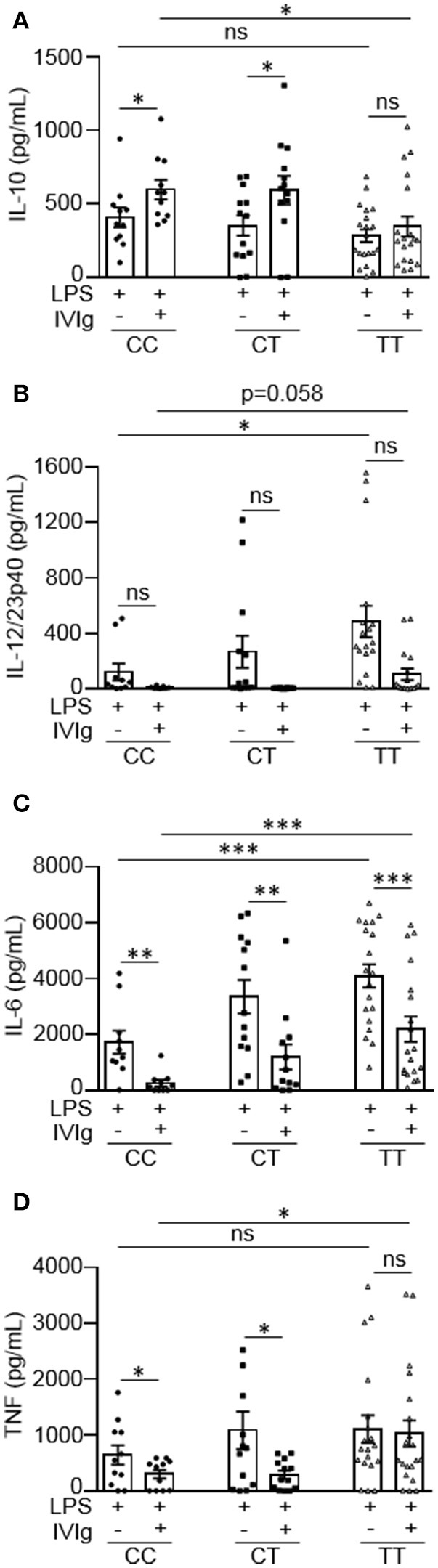
Monocytes from people with the FcγRIIA disease associated gene variant have lower IVIg-mediated anti-inflammatory responses to LPS. Monocytes from healthy control participants were stimulated with LPS (100 ng/ml) or [IVIg (5 mg/ml) + LPS (100 ng/ml)] for 24 h. Participants were genotyped for the FcγRIIA H131R polymorphism (rs1801274); CC = does not have the disease associated gene variant (low affinity), CT = heterozygous for the disease associated gene variant, and TT = homozygous for the disease associated gene variant (high affinity). Clarified cell supernatants were assayed for **(A)** IL-10, **(B)** IL-12/23p40, **(C)** IL-6, and **(D)** TNF and responses were stratified to genotype. Data are mean ± SEM from *n* = 11 CC participants, *n* = 13 CT participants, and *n* = 20 TT participants performed as independent experiments, assayed in duplicate. ^*^p < 0.05, ^**^p < 0.01, ^***^p < 0.001, and ns, not statistically significant. Statistical analyses were performed using a repeated measures one-way ANOVA with Dunn's multiple comparisons correction.

People with the FcγRIIA high affinity gene variant have increased susceptibility to inflammatory diseases, such as ulcerative colitis (16, 17). People with the risk variant have also been found to perform poorly on the antibody-based drug infliximab, which may work, in part, by activating macrophages to produce IL-10 (12, 23). Based on this, we wanted to investigate whether the FcγRIIA risk variant impacts monocyte anti-inflammatory responses to (IVIg + LPS). Monocytes from healthy control participants were unstimulated or stimulated with LPS, IVIg, or (IVIg + LPS). Participants were genotyped for the FcγRIIA H131R polymorphism (rs1801274) and cytokine production was stratified to genotype. People with the CC genotype do not have the disease associated gene variant, CT genotype are heterozygous for the risk variant, and the TT genotype are homozygous for the high affinity, disease associated gene variant. Unstimulated monocytes or monocytes stimulated with IVIg alone do not produce IL-10, IL-12/23p40, IL-6, or TNF (data not shown). IVIg co-treatment significantly increased LPS-induced IL-10 production in monocytes from people with the non-risk variant (CC) or people, who were heterozygous for the risk variant (CT), but did not significantly increase IL-10 production from people with the risk variant genotype (TT; Figure 5A). Genotype did not affect IL-10 production in response to LPS alone (Supplemental Figure 4A). This suggests that the risk variant compromises (IVIg + LPS)-induced IL-10 production.

Monocytes from people with the risk variant genotype (TT) had a reduced ability to limit pro-inflammatory cytokine production with IVIg and produced higher amounts of pro-inflammatory cytokines in response to LPS or (IVIg + LPS), compared to monocytes with the non-risk variant (Figures 5B–D). Monocytes from people with the risk variant genotype (TT) produced significantly higher amounts of IL-12/23p40 when stimulated with LPS and monocytes from some individuals with the risk variant maintained the ability to produce IL-12/23p40 when stimulated with (IVIg + LPS), which was ablated in monocytes from people with the non-risk variant (Figure 5B). Monocytes from participants with each of the gene variants were able to significantly reduce LPS-induced IL-6 production with IVIg co-treatment (Figure 5B). However, IL-6 production was significantly higher in both LPS and (IVIg + LPS)-stimulated monocytes from participants with the risk variant genotype (TT) compared to monocytes from people with the non-risk variant (Figure 5C). Monocytes heterozygous for the risk variant produced an intermediate amount of IL-6 when stimulated with LPS or (IVIg + LPS). Monocytes from people with the non-risk variant significantly decreased LPS-induced TNF production with IVIg co-treatment (53%), whereas monocytes from people with the risk variant did not (Figure 5D). Monocytes from participants with the risk variant genotype (TT) did not have significantly higher TNF production in response to LPS (*p* = 0.27), but produced significantly more TNF in response to (IVIg + LPS; Figure 5D). Monocytes heterozygous for the risk variant produced an intermediate amount of TNF when stimulated with LPS, had lower TNF production when stimulated with (IVIg + LPS), and had significantly reduced LPS-induced TNF production when co-treated with IVIg. Supplemental Figures 4B–D show pro-inflammatory cytokine production in response to LPS or (IVIg + LPS) graphed comparing genotypes. Of note, monocytes from people with the non-risk or risk variant reduced LPS-induced pro-inflammatory cytokine production to the same extent with (rhIL-10 + LPS) co-stimulation, demonstrating that monocytes from people with the risk variant do not have reduced responsiveness to IL-10 (Supplemental Figure 5). These results suggest that monocytes from people with the TT risk variant genotype are less able to limit pro-inflammatory cytokine production in response to IVIg because they produce lower amounts of IL-10.

### The high affinity FcγRIIA (TT) prevents (IVIg + LPS)-induced IL-10 production

**Figure 6:**
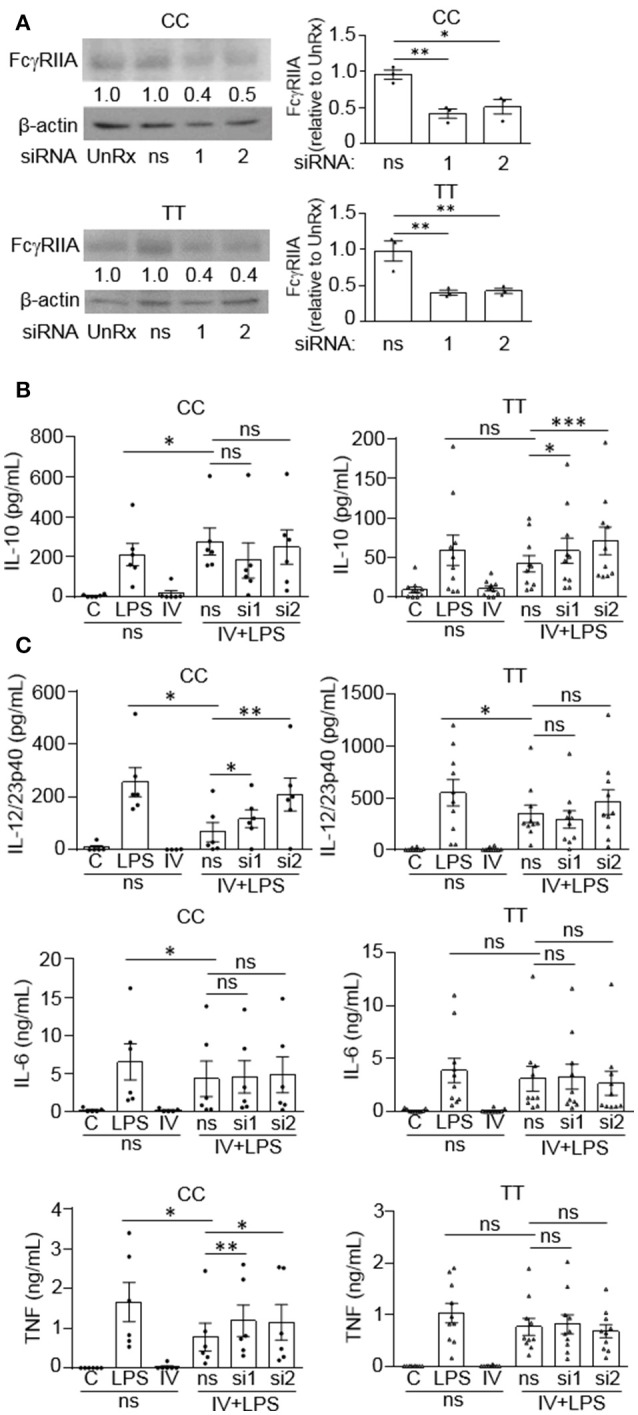
FcγRIIA prevents IVIg-induced IL-10 production in monocytes from people with the disease-associated gene variant. Monocytes from healthy control participants of the non-risk genotype (CC) and risk genotype (TT) were untreated (UnRx) or pre-treated for 48 h with a non-silencing siRNA (ns) or 2 different siRNAs to the FcγRIIA (si1 or si2). **(A)** Cell lysates (2.5 × 10^5^ cells / treatment) were prepared, separated by SDS-PAGE, analyzed by western blotting with antibodies for FcγRIIA and β-actin, as a loading control. Results are representative of *n* = 6 experiments for the non-risk genotype (CC) and *n* = 10 experiments for the risk genotype (TT); Monocytes were derived from 1 participant for each independent experiment. Densitometry for FcγRIIA normalized to β-actin and relative to the control (UnRx) are averaged and shown below each band. **(B,C)** Monocytes pre-treated with the ns siRNA control were unstimulated [control (C)] or stimulated with LPS (100 ng/ml), IVIg (5 mg/ml), or both, for 24 h, and monocytes pre-treated with FcγRIIA si1 and si2 were stimulated with IVIg (5 mg/ml) + LPS (100 ng/ml). Clarified cell supernatants were assayed for **(B)** IL-10, **(C)** IL-12/23p40, IL-6, and TNF. Data are mean ± SEM and are representative of *n* = 6 experiments for the non-risk genotype (CC) and *n* = 10 experiments for the risk genotype (TT). Monocytes were derived from 1 participant for each independent experiment and assayed in duplicate. ^*^p < 0.05, ^**^p < 0.01, ^***^p < 0.001, and ns = not statistically different for the comparisons indicated. Statistical analyses were performed using a repeated measures one-way ANOVA with Dunn's multiple comparisons correction.

Since we observed that monocytes from people with the disease associated gene variant have reduced anti-inflammatory responses to IVIg, we wanted to determine whether the FcγRIIA plays a direct role in reducing IL-10 production. Monocytes were genotyped and untreated (UnRx) or pre-treated with a non-silencing siRNA control (ns) or two siRNAs to the FcγRIIA (si1 and si2). Cell lysates were prepared, separated by SDS-PAGE, and analyzed by western blotting by probing with antibodies for the FcγRIIA and β-actin, as a loading control. The untreated and ns siRNA had similar levels of FcγRIIA in both genotypes (Figure 6A). The siRNA1 and 2 knocked down the FcγRIIA protein to a similar extent in monocytes harboring the non-risk and risk variants, 56 and 59% for siRNA1 and 46 and 56% for siRNA2 (Figure 6A). To determine the effect of reduced FcγRIIA expression on anti-inflammatory responses, siRNA pre-treated monocytes were unstimulated or stimulated with LPS, IVIg, or both. (IVIg + LPS)-stimulated monocytes from people with the non-risk variant genotype (CC) did not have increased IL-10 production when FcγRIIA was knocked down (Figure 6B, left). In contrast, (IVIg + LPS)-stimulated monocytes from people with the risk variant genotype (TT) had significantly increased IL-10 production with siRNA1 and with siRNA2, compared to the ns siRNA control (39% increase si1, 68% increase si2; Figure 6B, right).

FcγRIIA siRNAs increased production of pro-inflammatory cytokines by (IVIg + LPS)-stimulated monocytes from people with the non-risk variant, but not by monocytes from people with the risk variant (Figure 6C). Specifically, IL-12/23p40 was increased significantly with siRNA1 and 2 in the non-risk variant monocytes. (1.8-fold for siRNA1, 3.1-fold for siRNA2; Figure 6C, top left). However, IL-12/23p40 production was not significantly increased by FcγRIIA knockdown in monocytes from people with the risk variant (Figure 6C, top right). IL-6 production was not increased by FcγRIIA knockdown in either genotype (Figure 6C, middle). In monocytes from people with the non-risk variant, (IVIg + LPS)-stimulated TNF production increased significantly with siRNA1 and with siRNA2, compared to the ns siRNA (1.5-fold for si1 and si2; Figure 6C, bottom left). In contrast, there was no increase in (IVIg + LPS)-induced TNF production in monocytes from people with the risk variant when the FcγRIIA was knocked down (Figure 6C, bottom right). Taken together, these results suggest that the high affinity FcγRIIA risk variant (TT) blocks IVIg-induced IL-10 production, but it does not impact pro-inflammatory cytokine production directly. In contrast, the low affinity non-risk variant genotype (CC) limits (IVIg + LPS)-induced pro-inflammatory cytokine production.

### Monocytes from people with the FcγRIIA risk variant have dysregulated IVIg-induced MAPK phosphorylation

**Figure 7:**
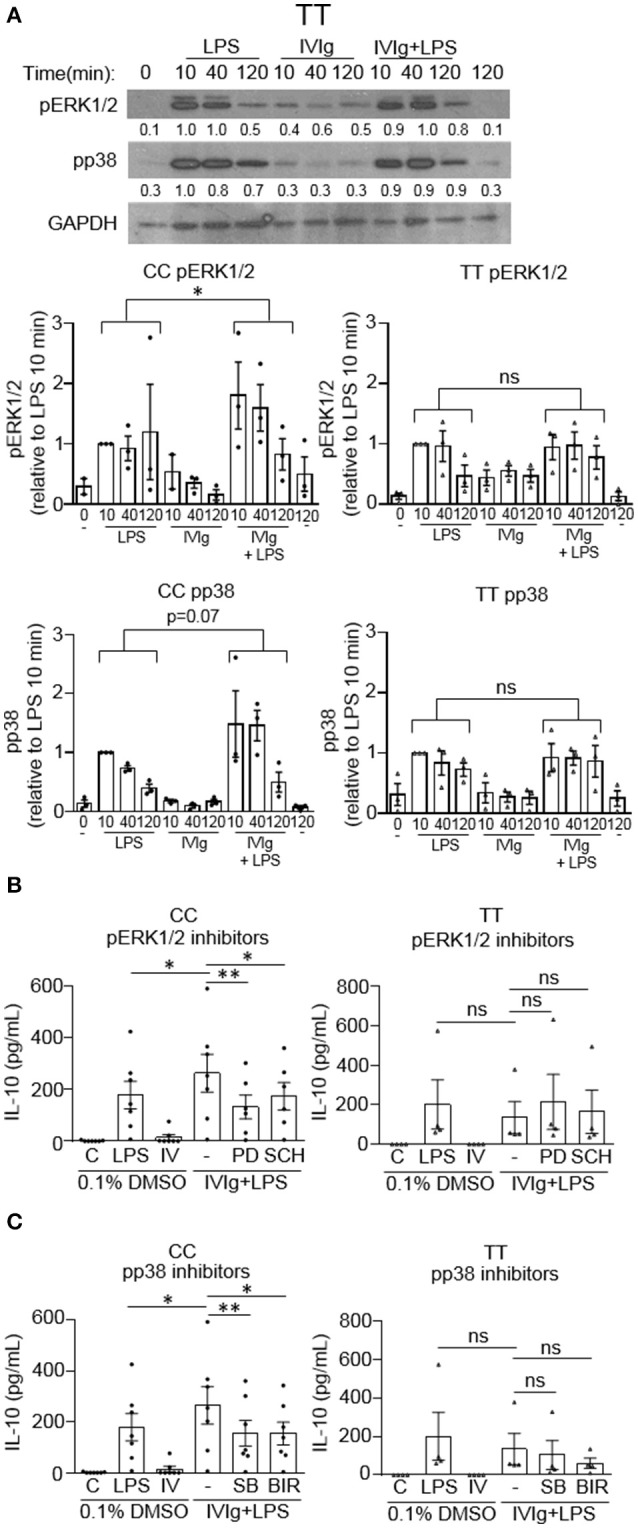
(IVIg + LPS)-induced MAPK phosphorylation is lower in monocytes from people with the FcγRIIA risk variant. **(A)** Monocytes from healthy participants with the non-risk genotype (CC) or the risk genotype (TT) were unstimulated or stimulated with LPS (100 ng/ml), IVIg (5 mg/ml), or both, for 0, 10, 40, or 120 min. Cell lysates (2.5 × 10^5^ cells / time point) were prepared at the indicated times. Lysates were separated by SDS-PAGE and analyzed by western blotting using phosphospecific antibodies for ERK1/2, p38, and using GAPDH, as a loading control. Representative western blot from participants with the TT genotype are shown in **(A)**. Results are representative of *n* = 3 experiments per genotype; monocytes were derived from 1 participant for each of 3 independent experiments. Densitometry for pERK1/2 and pp38; normalized to GAPDH and relative to LPS 10 min; are averaged from *n* = 3 independent experiments and shown below each band and values are graphed as mean ± SEM for each genotype. Monocytes were pre-treated for 1 h with an appropriate volume of DMSO, as a vehicle control, or **(B)** the ERK1/2 inhibitors, PD98059 (PD) and SCH772984 (SCH); or **(C)** the p38 inhibitors, SB203580 (SB) and BIRB796 (BIR) and were unstimulated **(C)** or stimulated with LPS (100 ng/ml), IVIg (5 mg/ml), or both for 24 h. Clarified cell supernatants were analyzed for IL-10 by ELISA. **(B,C)** Values are reported as mean ± SEM for *n* = 7 participants for the CC genotype and *n* = 4 participants for the TT genotype performed as independent experiments, and assayed in duplicate. ^*^p < 0.05, ^**^p < 0.01, and ns = not statistically different for the comparisons indicated. Statistical analyses were performed using a two-way ANOVA in **(A)** and repeated measures one-way ANOVA in **(B,C)** with Dunn's multiple comparisons correction.

We have shown that MAPK activation is required for (IVIg + LPS) induced IL-10 production. Since IL-10 production is compromised in people with the high affinity disease associated FcγRIIA gene variant, we next asked whether this was due to a failure to activate MAPKs. Monocytes with either the non-risk or risk variant were unstimulated (0 and 120 min) or stimulated with LPS (100 ng/ml), IVIg (5 mg/ml), or both for 10, 40, or 120 min. Cell lysates from either genotype were analyzed by western blot for pERK1/2, pp38, and GAPDH, as a loading control. A representative western blot for the non-risk variant is shown and described in Figure 3A, and for the risk variant is shown and described in Figure 7A. Non-risk and risk variant western blot quantifications for *n* = 3 subjects per variant are graphed in Figure 7A. ERK1/2 and p38 phosphorylation were induced in response to stimulation with LPS in monocytes from people with the risk variant, but were not significantly increased by concomitant treatment with IVIg, as for people with the non-risk variant (Figure 7A). In monocytes from people with the risk variant, ERK1/2 activation did not significantly increase upon stimulation with (IVIg +LPS) compared to LPS at 10 or 40 min and was only modestly higher at 120 min (Figure 7A). Similarly, monocytes from people with the risk variant did not have increased and prolonged p38 activation upon stimulation with (IVIg + LPS) compared to stimulation with LPS alone (Figure 7A).

Because MAPK activation was not induced by (IVIg + LPS) relative to either IVIg or LPS alone in monocytes from people with the FcγRIIA risk variant, we asked whether IL-10 production was MAPK-dependent. Monocytes were genotyped and unstimulated or stimulated with LPS, IVIg, or (IVIg + LPS) in the absence or presence of inhibitors for MAPKs, ERK1/2 or p38. For ERK1/2 inhibition; in monocytes from people with the non-risk variant, PD98059 (PD), and SCH772984 (SCH) significantly reduced IL-10 production in response to (IVIg + LPS) compared to the DMSO control (50 and 34%, respectively; Figure 7B, left). In contrast, monocytes from people with the risk variant did not have a statistically significant decrease in IL-10 production when ERK1/2 was inhibited. Compared to the solvent control (DMSO), both p38 inhibitors SB203580 (SB) and BIRB796 (BIR) significantly reduced (IVIg + LPS)-induced IL-10 production in monocytes from people with the non-risk variant (41% SB, 42% BIR; Figure 7C, left), whereas modest reductions in monocytes from people with the risk variant did not reach significance (23% SB, *p* = 0.07; 55% BIR, *p* = 0.07; Figure 7C, right). These results suggest that monocytes from people with the high affinity FcγRIIA risk variant fail to induce robust MAPK activation upon stimulation with (IVIg + LPS), which may underlie their defect in (IVIg + LPS)-induced IL-10 production.

## Discussion

IVIg exerts its anti-inflammatory activity, at least in part, by production of IL-10, which is compromised in monocytes from people with the FcγRIIA risk variant. IL-10 limits immune responses to prevent damage to the host, by negatively regulating production of TLR-induced pro-inflammatory cytokines, antigen presentation, and activation of CD4+ T cells (4). Clinical trials using recombinant human IL-10 show only modest improvement for Crohn's disease and RA, partially due to systemic rather than cite specific administration (3). Our study is the first to report that IVIg increases IL-10 production in human monocytes, which could allow for IVIg primed monocytes/macrophages to produce IL-10 directly at sites of inflammation when they are activated. IVIg treatment has similarly been reported to increase IL-10 production in human dendritic cells *in vitro* (24). In contrast, IVIg has been reported to reduce LPS-induced IL-10 production in macrophage colony-stimulating factor (MCSF)-derived human macrophages *in vitro*, however the experimental conditions differed from our study and the FcγRIIA genotype of the macrophages described is not known (25). *In vivo*, IL-10 is increased in serum and IL-10 production is increased by PBMCs from ITP patients post IVIg treatment (26, 27). Other antibodies can also induce IL-10 production. Human M(Ic) and M(infliximab), activated with the anti-TNF IgG drug, infliximab; increase IL-10 production in response to pro-inflammatory stimuli *in vitro* (23, 28, 29). Moreover, it has been suggested that infliximab activates intestinal macrophages to an anti-inflammatory or “regulatory” phenotype *in vivo* specifically in treatment-responsive IBD patients, but not in non-responders (30). In RA patients, people with the FcγRIIA risk variant are less responsive to the anti-TNFα drugs, infliximab and adalimumab, compared to people with the non-risk variant suggesting that the FcγRIIA genotype may be useful in predicting responses to therapy (12, 31). Nine to 23% of Kawasaki disease patients are not responsive to IVIg therapy and up to 40% of IBD patients are, or will become, refractory to anti-TNFα therapy, approximately half of whom have not developed anti-drug antibodies (32, 33). Further investigation is required to determine whether the FcγRIIA gene variant (rs1801274) genotype can be used in a precision medicine approach to predict responsiveness to IVIg as well as other antibody-based biological therapies.

We have found that IVIg reduces human monocyte production of LPS-induced pro-inflammatory cytokines IL-12/23p40, IL-6, and TNF, and reduction of these cytokines is impaired in monocytes from people with the FcγRIIA risk variant. LPS-induced IL-12, IL-6, and TNFα production have similarly been reported to decrease in human dendritic cells, monocytes, or granulocyte-macrophage colony-stimulating factor (GM-CSF)-derived macrophages, respectively, treated with IVIg (24, 25, 34, 35). Reduced IL-6 production by M(IVIg + LPS) is similar to that reported for macrophages co-stimulated with immune complexes and LPS [M(Ic + LPS)], however, M(Ic + LPS) do not reduce IL-12/23p40 and TNF production (28). Our data are also consistent with *in vivo* observations wherein IVIg treatment reduces serum IL-6 and IL-6 production by LPS-stimulated whole blood in children with Kawasaki disease, and reduces serum TNF and IL-1β levels in patients with Guillain-Barré syndrome (36, 37). Few studies exist linking the FcγRIIA gene variant to antibody-mediated immune responses, but previous findings are consistent with our data. Mononuclear cells with the risk variant have higher production of IL-1β when activated with IgG_2_ (38). Patients with the risk variant receiving anti-D intravenous antibodies, which are antibodies to Rho(D) present on some RBCs, for ITP, have higher plasma levels of IL-6, TNFα, and MCP-1 post-infusion compared to patients with the non-risk variant (27). The reduction of potentially pathogenic pro-inflammatory cytokines, IL-12, IL-23, IL-6, and TNF, may be a unique characteristic of IVIg-induced immunosuppression, which is impaired in monocytes from people with the risk variant.

We have used two independent approaches, blocking antibodies and siRNA knockdown, to demonstrate that FcγRI and FcγRIIB, but not FcγRIIA or FcγRIII, are involved in (IVIg + LPS)-induced IL-10 production. FcγRI contains activating intracellular tyrosine-based activation motifs (ITAMs) on its cytoplasmic tail, whereas the FcγRIIB contains inhibitory intracellular tyrosine-based inhibitory motifs (ITIMs) on its cytoplasmic tail. FcγRI-mediated activation has been implicated in murine macrophage IL-10 production downstream of Ic (39). The FcγRIIB ITIM recruits the Src homology region 2 domain-containing phosphatase-1 and 2 (SHP-1 and 2) and the lipid phosphatase, SH2–containing inositol polyphosphate phosphatase (SHIP), to the cell membrane, the latter of which has been implicated in IL-10 production by regulatory B cells (40). Thus activating and inhibitory FcγRs may cooperate in human monocytes to promote anti-inflammatory (IVIg + LPS)-induced IL-10 production. *In vivo*, inhibitory signaling downstream of the FcγRIII [inhibitory ITAM (ITAMi) signaling] has been implicated in the IVIg-dependent reduction of TNFα and MCP-1 in a unilateral uretral obstruction nephritis mouse model; and FcγRIIB has been implicated in IVIg's anti-inflammatory activity in a mouse model of intracerebral hemorrhage, although IL-10 levels were not measured (41, 42). In whole blood from healthy humans, FcγRI and FcγRIII are responsible for IVIg-induced IL-10 production in response to LPS, with larger reductions in IL-10 when both receptors are blocked (26). In human macrophages *in vitro*, infliximab can bind and act through FcγRI, and can promote IL-10 production in response to LPS (23, 43); and FcγRI, FcγRIIA, FcγRIII, and FcγRIIB, are all reported to contribute to IL-10 production by human macrophages activated with Ic and Pam3CSK4, a bacterial lipoprotein (44). Based on our data, strategies to cross-link FcγRI and FcγRIIB could be an effective replacement therapy for IVIg in macrophage-mediated diseases, and targeting these receptors directly is predicted to be independent of the FcγRIIA genotype.

We have shown that the FcγRIIA disease-associated gene variant limits both IL-10 production and reduction of pro-inflammatory cytokines by M(IVIg + LPS). This is the first study, to our knowledge, that directly shows the differential effects that this FcγRIIA genetic polymorphism has on monocyte IL-10 and pro-inflammatory cytokine production. The high affinity FcγRIIA risk variant could directly limit IVIg-induced IL-10 by sequestering IgG antibodies from FcγRI and FcγRIIB, which we have shown promote IVIg-induced IL-10 production. In addition, low level engagement of the non-risk FcγRIIA gene variant (low affinity) may drive ITAMi-mediated reduction of pro-inflammatory cytokine production, as is the case for the FcαRI and FcγRIII; whereas saturation of the high affinity FcγRIIA risk variant may promote ITAM activating signaling contributing to higher pro-inflammatory cytokine production (45, 46). In either scenario, blocking the high affinity FcγRIIA risk variant prior to IVIg treatment could increase FcγRI- and FcγRIIB-mediated IL-10 production and improve IVIg's anti-inflammatory activity in people, who harbor the high affinity FcγRIIA risk variant.

**Figure 8:**
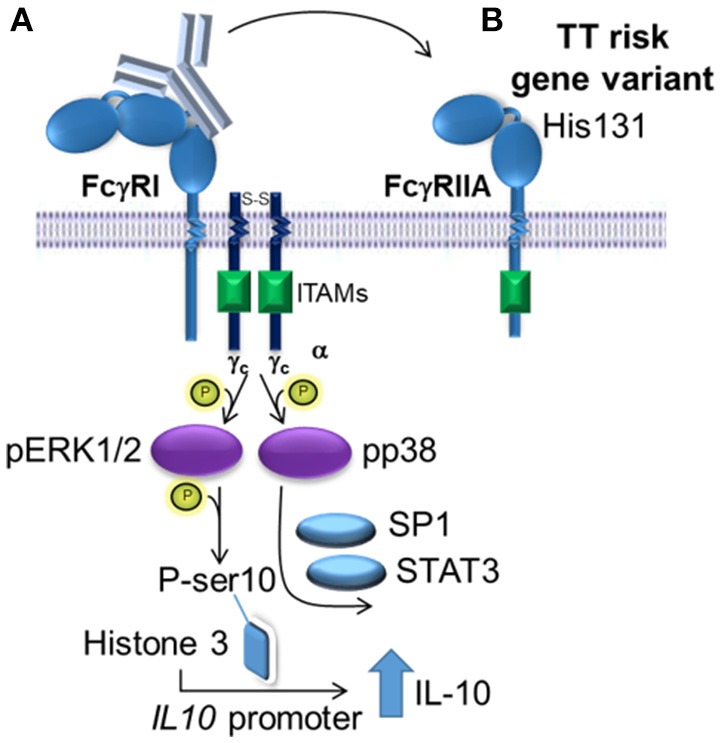
Proposed model of IVIg-induced IL-10 production in monocytes from people with the non-risk, low affinity FcγRIIA gene variant. **(A)** In monocytes from people with the low affinity, non-risk gene variant, the FcγRI induces increased IL-10 production in response to IVIg. ERK1/2 activation is increased, which primes cells for IL-10 production by phosphorylating ser10 on histone 3 opening up the *IL10* promoter and phosphorylating p38, which drives specificity protein 1 (SP1)- and signal transducer and activator of transcription 3 (STAT3)-mediated transcription of *IL10*. **(B)** The high affinity, risk variant FcγRIIA sequesters IVIg antibodies from the FcγRI in monocytes, which prevents activation of ERK1/2 and p38, and limits IL-10 production.

The activation of the MAPKs, ERK1/2 and p38, were required for (IVIg + LPS)-induced IL-10 production in monocytes from people with the non-risk variant, as siRNA knockdown and two distinct pharmacological inhibitors for each of ERK1/2 and p38, reduced IL-10 production. M(Ic +LPS) require FcγRI, which actives Erk1/2 and leads to phosphorylation of serine 10 on histone 3 opening up the *il10* promoter, while p38 activation drives Sp1- and STAT3-mediated transcription of IL-10 (21, 47). Our data supports a similar model for human M(IVIg + LPS), in which ERK1/2 is activated modestly in response to IVIg along and robustly in response to (IVIg +LPS), priming monocytes for IL-10 production; while p38 is not activated by IVIg and robustly activated by co-stimulation with (IVIg + LPS) driving *IL10* transcription (21, 47). Macrophages' ability to elicit stronger ERK1/2 activation than seen in dendritic cells, leads to their ability to produce higher amounts of IL-10 (48). Indeed, in murine M(Ic + LPS), the stronger the FcγRI signaling and Erk1/2 activation, the higher the IL-10 production (47, 48). Human monocytes treated with LPS and ethanol have increased IL-10 production, which has been shown to be driven by increased activation of p38 (49). Interestingly, pharmacological inhibition of MAPKs did not block IL-10 production by monocytes from people with the risk variant and MAPK activation was not enhanced by (IVIg + LPS) compared to LPS alone in monocytes from people with the FcγRIIA risk variant. This data is consistent with our model suggesting that the FcγRIIA risk variant sequesters antibodies from FcγRI and perhaps FcγRIIB, preventing downstream activation of MAPKs required for IL-10 production (Figure 8).

In conclusion, our study shows that IVIg can induce anti-inflammatory responses in human monocytes, by increasing IL-10 production and reducing pro-inflammatory cytokine production in response to LPS. This could explain why IVIg is effective for a diverse range of inflammatory and autoimmune diseases, as IL-10 has a critical, non-redundant role in turning off both innate and adaptive immune signaling and restoring tissue homeostasis. IL-10 production by monocytes may not be the only mechanism for IVIg's anti-inflammatory activity but may cooperate with other IVIg-mediated anti-inflammatory activities (50, 51). Monocytes from people with the high affinity FcγRIIA risk variant have lower anti-inflammatory IL-10 production and higher pro-inflammatory cytokine production in response to LPS, which may contribute to increased risk of developing inflammatory diseases. In addition, monocytes from people with the high affinity FcγRIIA risk variant are less able to induce IL-10 production and limit pro-inflammatory cytokine production in response to (IVIg + LPS) than monocytes from people with the non-risk variant, which may contribute to poor responses to IVIg and antibody-based biological therapy. This novel mechanism of action for IVIg suggests that genotyping the FcγRIIA gene variant (rs1801274), alone or in combination with additional bioassays, may be useful in predicting responsiveness to IVIg, and perhaps other antibody-based biological therapies. In addition, blocking the high affinity FcγRIIA in people with the risk variant, could improve their response to IVIg. Finally, the results of this study may be useful to develop new therapeutic strategies to replace IVIg by cross-linking FcγRIs and FcγRIIBs to promote anti-inflammatory monocyte activation in monocyte/macrophage-mediated diseases, which is predicted to be independent of the FcγRIIA genotype.

## Datasets are available on request

The raw data supporting the conclusions of this manuscript will be made available by the authors, without undue reservation, to any qualified researcher.

## Author contributions

LK and LS conceived of the study and the experiments. LK and LS designed the experiments. LK, SM, ZZ, TV, KH, KD, and KS performed the experiments and data analysis. ST supervised KB. LS supervised all aspects of the study. LK and LS prepared and wrote the manuscript with contributions from all authors.

### Conflict of interest statement

The authors declare that the research was conducted in the absence of any commercial or financial relationships that could be construed as a potential conflict of interest.
