# Supplementary material for: IVIg and LPS Co-stimulation Induces IL-10 Production by Human Monocytes, Which Is Compromised by an FcγRIIA Disease-Associated Gene Variant
Source: Front Immunol. 2018 Nov 20;9:2676. doi: 10.3389/fimmu.2018.02676 (PMC6255983; doi:10.3389/fimmu.2018.02676)
Supplement: Supplementary file 1 [file Presentation_1.pdf]

Supplementary Material

IVIg and LPS Co-stimulation Induces IL-10 Production By Human Monocytes, Which Is Compromised By An FcγRIIA Disease-Associated Gene Variant

Lisa K. Kozicky,[1] Susan C. Menzies,[1] Zheng Yu Zhao,[1] Tariq Vira,[1] Kiera Harnden,[1] Kwestan Safari,[1] Kate L. Del Bel,[2] Stuart E. Turvey,[2] and Laura M. Sly[1*]

*** Correspondence:** Laura M. Sly laurasly@mail.ubc.ca

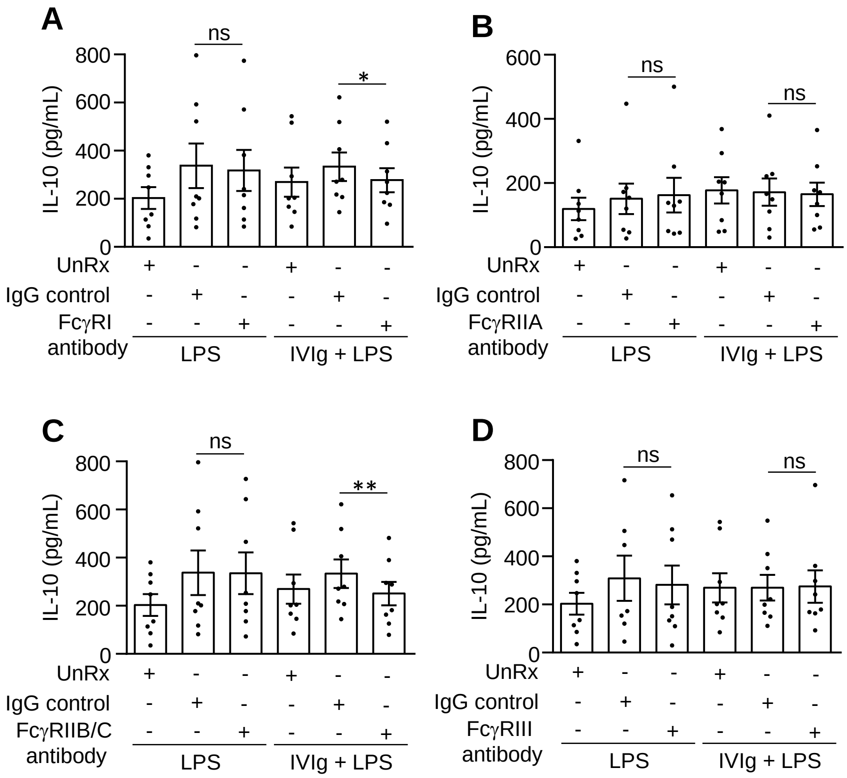

Supplemental Figure 1. FcγRI and FcγRIIB are required for IVIg-induced IL-10 production in response to LPS. Monocytes were untreated (UnRx) or pre-treated for 1 h with an IgG isotype control or a blocking antibody against (**A**) FcγRI (100 μg/mL), (**B**) FcγRIIA (50 μg/mL), (**C**) FcγRIIB/C (100 μg/mL), or (**D**) FcγRIII (50 μg/mL). Cells were stimulated with LPS (100 ng/mL) or (IVIg (5 mg/mL) + LPS (100 ng/mL)) for 24 h. Clarified cell supernatants were assayed for IL-10. Monocytes were derived from 1 participant for each of 8 independent experiments, and were assayed in duplicate. *$p < 0.05$, **$p < 0.01$ and ns = not statistically different. Statistical analyses were performed using a repeated measures one-way ANOVA with Dunn's multiple comparisons correction.

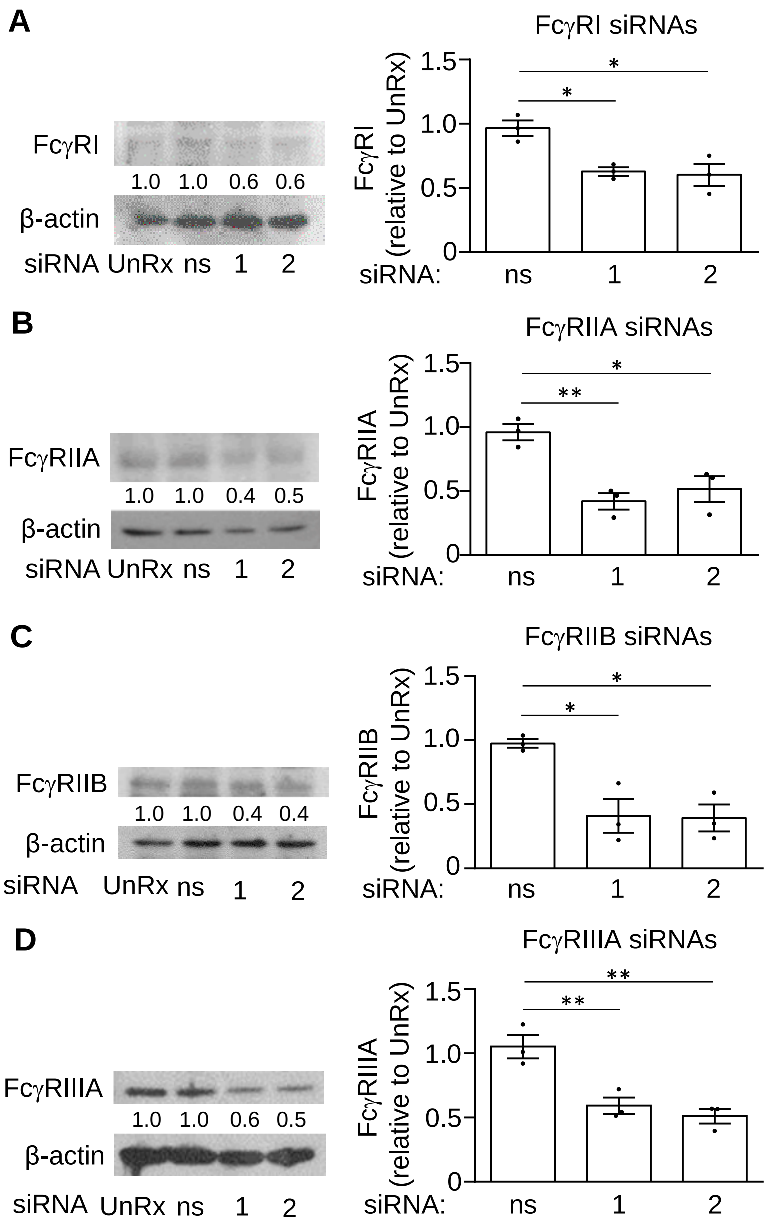

Supplemental Figure 2. FcγRI, FcγRIIA, FcγRIIB, and FcγRIIIA expression is reduced with siRNAs. Monocytes were untreated (UnRx) or pre-treated for 48 h with a non-silencing siRNA (ns) or 2 different siRNAs (si1 or si2) to the FcγRI (**A**), FcγRIIA (**B**), FcγRIIB (**C**), or FcγRIIIA (**D**). Cell lysates were prepared, separated by SDS-PAGE and analyzed by western blotting using antibodies to each receptor and β-actin, as a loading control. Results are from $n = 3$ experiments; monocytes were derived from 1 participant for each of 3 independent experiments. Densitometry for receptor expression normalized to β-actin and relative to untreated control (UnRx); are averaged from $n = 3$ independent experiments and are shown as mean ± SEM. *$p < .05$ and **$p < .01$. Statistical analyses were performed using a one-way ANOVA with Dunnett's multiple comparisons correction.

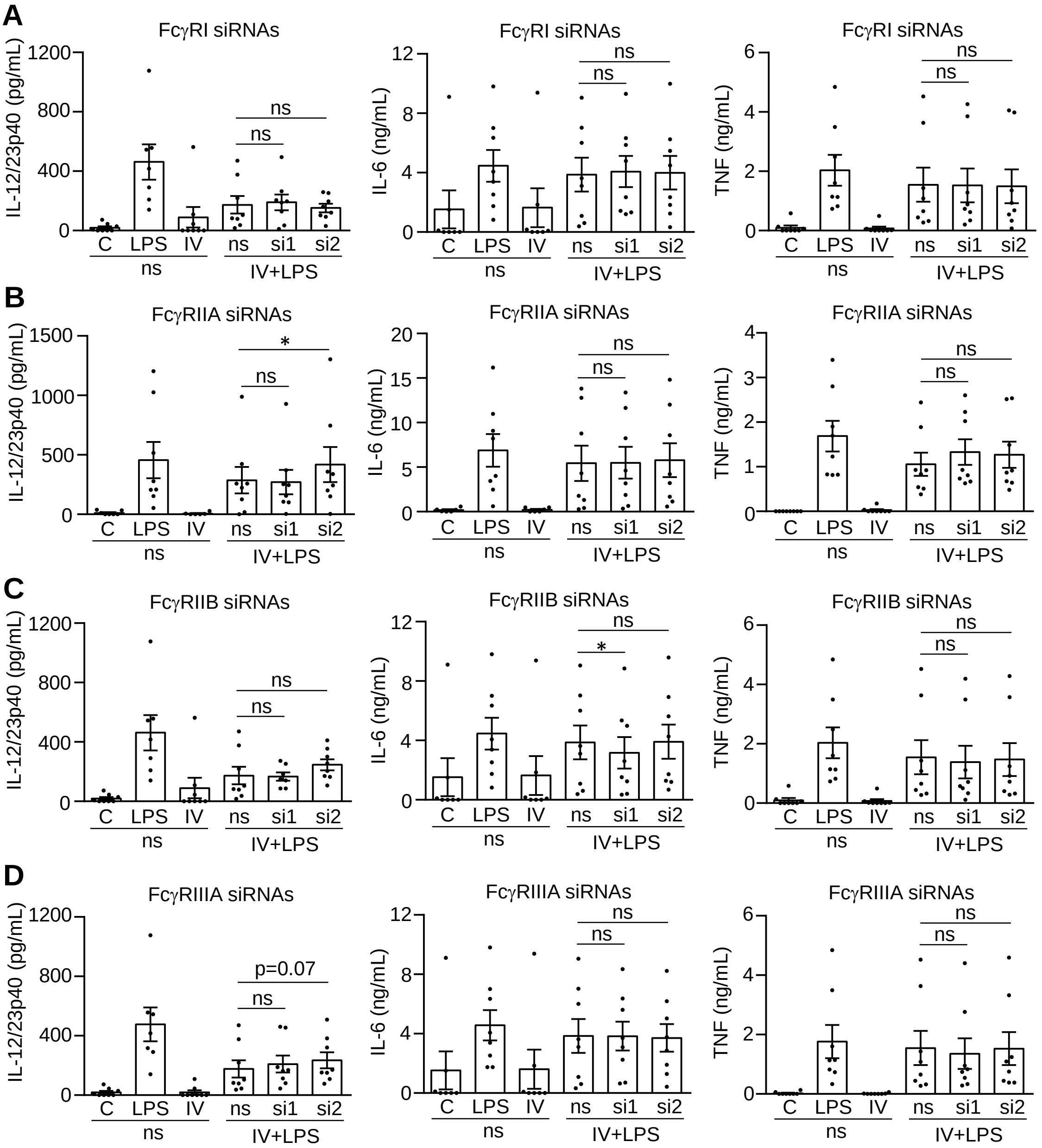

Supplemental Figure 3. FcγRI, FcγRIIA, FcγRIIB, or FcγRIIA are not required for (IVIg + LPS)-induced reduction of pro-inflammatory cytokines. Monocytes were pre-treated for 48 h with a non-silencing siRNA (ns) or 2 different siRNAs (si1 or si2) to the FcγRI (**A**), FcγRIIA (**B**), FcγRIIB (**C**), or FcγRIIIA (**D**). Monocytes pre-treated with the ns siRNA control were unstimulated (control (C)) or stimulated with LPS (100 ng/mL), IVIg (5 mg/mL), or both, for 24 hours, while the monocytes pre-treated with si1 or si2 were stimulated with IVIg (5 mg/mL) + LPS (100 ng/mL). Clarified cell supernatants were assayed for IL-12/23p40, IL-6, and TNF. Data are mean ± SEM. Results are representative of $n = 8$ experiments; monocytes were derived from 1 participant for each of 8 independent experiments, and were assayed in duplicate. *$p < 0.05$ and ns = not statistically different. Statistical analyses were performed using a repeated measures one-way ANOVA with Dunn's multiple comparisons correction.

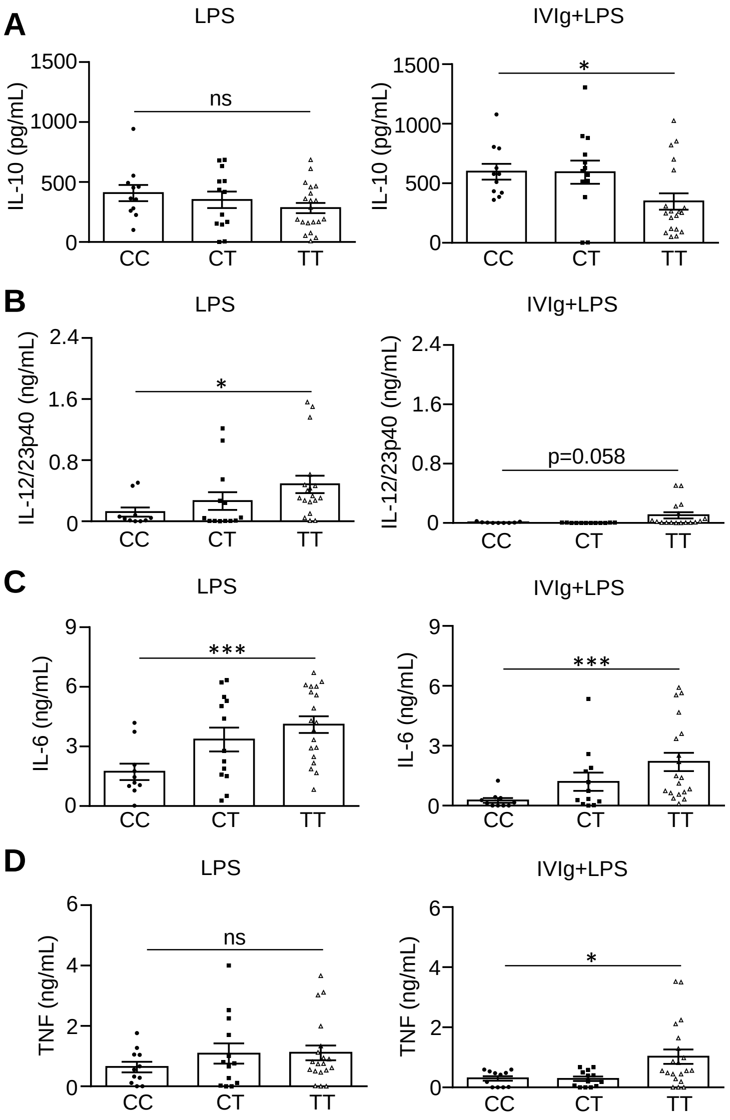

Supplemental Figure 4. Monocytes from people with the FcγRIIA disease associated gene variant have lower anti-inflammatory responses to (IVIg+LPS). Monocytes from healthy control participants were stimulated with LPS (100 ng/mL) or (IVIg (5 mg/mL) + LPS (100ng/mL)) for 24 h. Participants were genotyped for the FcγRIIA H131R polymorphism (rs1801274); CC = does not have the disease associated gene variant (low affinity), CT = heterozygous for the disease associated gene variant, and TT = homozygous for the disease associated gene variant (high affinity), and data were stratified based on genotype. Clarified cell supernatants were assayed for (**A**) IL-10, (**B**) IL-12/23p40, (**C**) IL-6, and (**D**) TNF. Data are mean ± SEM from n = 11 CC participants, n = 13 CT participants, and n = 20 TT participants performed as independent experiments, assayed in duplicate. *p < .05, **p <.001, and ns = not statistically significant. Statistical analyses were performed using non-parametric, unpaired t-tests.

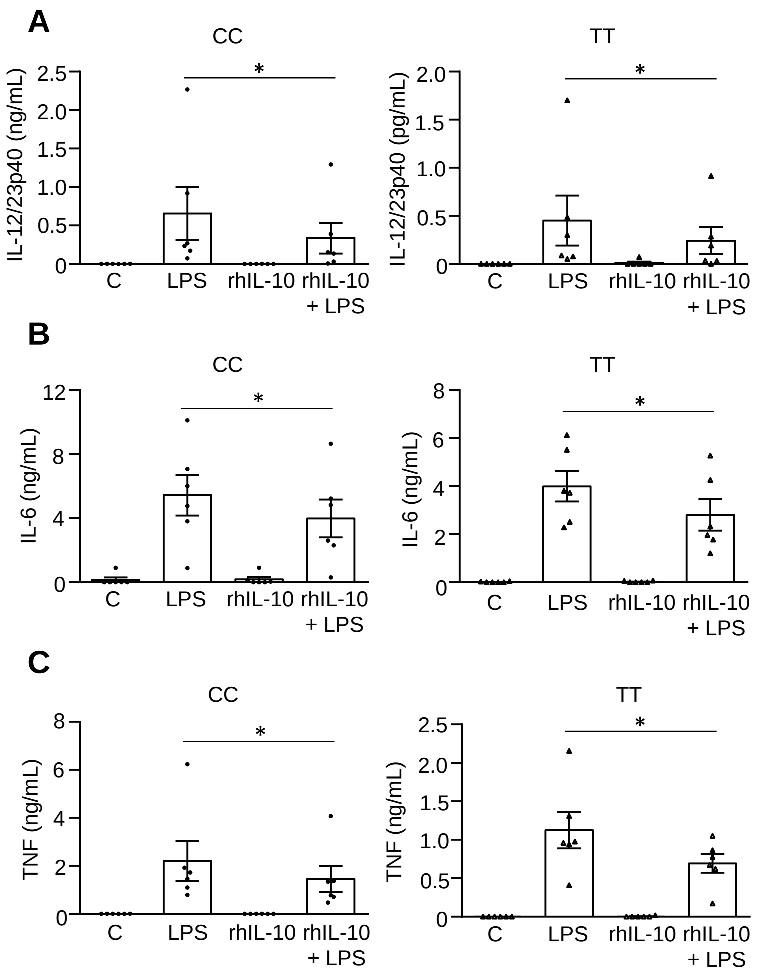

Supplemental Figure 5. IL-10 reduced LPS-induced pro-inflammatory cytokine production by monocytes from people with either FcγRIIA gene variant. Monocytes from healthy control participants with the non-risk genotype (CC) and risk genotype (TT) were untreated (C) or stimulated with LPS (100 ng/mL), recombinant human IL-10 (rhIL-10) (400 pg/mL) or (rhIL-10 (400 pg/mL) + LPS (100 ng/mL)) for 24 h. Clarified cell supernatants were assayed for (**A**) IL-12/23p40, (**B**) IL-6, and (**C**) TNF. Data are mean ± SEM from *n* = 6 participants per genotype, performed as independent experiments, and assayed in duplicate. *$p < 0.05$ for the comparisons indicated. Statistical analyses were performed using a non-parametric, paired *t*-test.
